# Real-World Data on the Usage of Hemopatch® as a Hemostat and Dural Sealant in Cranial and Spinal Neurosurgery

**DOI:** 10.7759/cureus.34387

**Published:** 2023-01-30

**Authors:** Karl-Michael Schebesch, Tomas Hrbac, Radim Jančálek, Lukas Krska, Javier Marquez-Rivas, Peter Solar

**Affiliations:** 1 Department of Neurosurgery, University Medical Centre of Regensburg, Regensburg, DEU; 2 Department of Neurosurgery, Faculty Hospital Ostrava, University of Ostrava, Ostrava, CZE; 3 Department of Neurosurgery, St. Anne’s University Hospital and Faculty of Medicine, Masaryk University, Brno, CZE; 4 Department of Pediatric Surgery, Hospital Universitario Virgen del Rocío, Sevilla, ESP

**Keywords:** hemopatch®, watertight closure, csf fistula, csf leakage, dura mater, supratentorial, infratentorial, cranial, spinal, neurosurgery

## Abstract

Background and objectives

Cerebrospinal fluid (CSF) leakage is a significant complication in cranial and spinal interventions. Hemostatic patches such as Hemopatch^®^ are therefore used to support the watertight closure of the dura mater. Recently, we published the results of a large registry documenting the effectiveness and safety of Hemopatch^®^ in various surgical specialties, including neurosurgery. Here we aimed to analyze the outcomes from the neurological/spinal cohort of this registry in more detail.

Methods

Based on the data from the original registry, we performed a post hoc analysis for the neurological/spinal cohort. The Hemopatch^®^ registry was designed as a prospective, multicenter, single-arm observational study. All surgeons were familiar with the application of Hemopatch^®^ and it was used at the discretion of the responsible surgeon. The neurological/spinal cohort was open for patients of any age if they had received Hemopatch^®^ during an open or minimally invasive cranial or spinal procedure. Patients with known hypersensitivity to bovine proteins or brilliant blue, intraoperative pulsatile severe bleeding, or an active infection at the potential target application site (TAS) were excluded from the registry. For the posthoc evaluation, we stratified the patients of the neurological/spinal cohort into two sub-cohorts: cranial and spinal. We collected information about the TAS, intraoperative achievement of watertight closure of the dura, and occurrence of postoperative CSF leaks.

Results

The registry comprised 148 patients in the neurological/spinal cohort when enrolment was stopped. The dura was the application site for Hemopatch^®^ in 147 patients (in one patient in the sacral region after tumor excision), of which 123 underwent a cranial procedure. Twenty-four patients underwent a spinal procedure. Intraoperatively, watertight closure was achieved in 130 patients (cranial sub-cohort: 119; spinal sub-cohort: 11). Postoperative CSF leakage occurred in 11 patients (cranial sub-cohort: nine; spinal sub-cohort: two). We observed no serious adverse events related to Hemopatch^®^.

Conclusion

Our post hoc analysis of real-world data from a European registry confirms the safe and effective use of Hemopatch^®^ in neurosurgery, including cranial and spinal procedures, as also observed in some case series.

## Introduction

Cerebrospinal fluid (CSF) leakage is a relevant complication in cranial and spinal interventions, increasing the risk of infections and the duration of hospital stays [1]. In cranial procedures, CSF leaks have been reported at rates from 0.9 to 34.6% [2-7]. In spinal surgery, CSF leakage rates from 0 to 11.7% have been observed [1,8,9]. In addition to traditional neurosurgical techniques, hemostatic patches such as Hemopatch^®^ can be helpful tools to achieve watertight closure of the dura mater in order to prevent leakage of CSF into the surrounding tissue [4,10].

Hemopatch^®^ is composed of a thin, pliable collagen pad coated with a protein-reactive polyethylene glycol monomer (pentaerythritol polyethylene glycol ether tetra-succinimidyl glutarate, NHS-PEG). The collagen pad has a high liquid absorption capacity, while the covalent binding of the NHS-PEG coating to proteins results in strong adherence to the underlying tissue. The polymerized NHS-PEG builds a hydrogel that allows rapid adhesion to the target site, where the patch forms a liquid-tight barrier. Additionally, blood clotting is promoted by the collagen matrix of the pad. Crosslinking is facilitated by blood and other body fluids or can be achieved with sodium bicarbonate solution if the Hemopatch^®^ is applied on a dry target application site [11].

Several small series previously described the effective and reliable use of Hemopatch^®^ in neurosurgery to reduce the incidence of CSF leakage [1,4,10,12-14].

Recently, data from a registry documenting the real-world clinical application of Hemopatch^®^ was published [15]. This registry was the largest carried out for this product so far and included cases from several surgical specialties such as hepatobiliary, pulmonary, neurological/spinal, urological, and general surgery. In summary, this registry documented the effectiveness and safety of Hemopatch^®^ in achieving hemostasis and sealing in various tissues, not only in open procedures but also in minimally invasive surgery [15]. Here we report on specific outcomes from the neurological/spinal cohort of this registry (n=148).

## Materials and methods

Study design and participants

Ethical approval for this study was obtained from the Ethics Commission of the University of Cologne (Nr. 17-344 dated 11/8/2017). Details of the study design and selection of participants were reported previously [15]. In brief, the registry was designed as a prospective, multicenter, single-arm, observational study. It was conducted between November 2017 and January 2019 at 23 study sites in six European countries (Austria, Czech Republic, Germany, Italy, Poland, and Spain). Four of these centers contributed cases to the neurological/spinal cohort. The surgeons were familiar with the application of Hemopatch^®^ and decided whether to use it or not according to the institutional standards.

Patients of any age were eligible for the neurological/spinal cohort if they had received Hemopatch^®^ during an open or minimally invasive cranial or spinal procedure. Known hypersensitivity to bovine proteins or brilliant blue, intraoperative pulsatile or severe bleeding at the potential target application site (TAS), or an active infection at the TAS were reasons for excluding the patient from the registry. If Hemopatch^®^ was applied, it was held in place for two minutes and, if needed according to the instructions for use, bicarbonate solution was used to moisten the TAS before placement of the pad [16]. The pad should overlap the tissue lesion by 1 cm in all directions, and if multiple pads are used, they should also overlap by 1 cm.

Perioperative data on the effectiveness and safety of Hemopatch^®^ as well as the potential use of bicarbonate was recorded with an electronic case report form (eCRF) [15].

Effectiveness endpoints and safety outcomes

Effectiveness endpoints as well as safety outcomes of the registry have already been reported [15].

Briefly, the primary endpoints for intraoperative effectiveness were the percentage of patients achieving hemostasis within two minutes and the percentage of patients achieving hemostasis without re-bleeding at the time of surgical closure. In the neurological/spinal cohort presented here, an additional intraoperative effectiveness endpoint in terms of leakage control was defined as the achievement of a watertight closure, i.e. no CSF leakage after inspection according to the local standard of care such as the Valsalva maneuver. The primary postoperative effectiveness endpoint for the neurological/spinal cohort was defined as the incidence of a postoperative CSF leakage presenting as an external or internal accumulation of CSF, including pseudo meningocele during the four-week follow-up period [15].

To assess the safety of Hemopatch^®^, the incidence of adverse events of special interest was defined as primary safety endpoints. These were as follows: allergic reaction to Hemopatch^®^, re-bleeding at the TAS, hematoma at the TAS, and/or local infections at the TAS [15].

Secondary safety endpoints included the number of Hemopatch^®^ units applied, use of bicarbonate in combination with Hemopatch^®^, need for intraoperative surgical revisions due to bleeding, air, or other body fluid leakage, intraoperative transfusions (number, type, and amount of blood product), surgery duration, postoperative transfusions up to 72 hours after surgery (number, type, and amount of blood product), days in the intensive care unit, length of hospital stay as well as the surgeon's product assessment and satisfaction, assessed by the surgeon´s questionnaire [15].

Statistical methods and data sets

As described previously in detail, statistical analysis of the Hemopatch^®^ registry was mainly descriptive and included sample size (n), mean, standard deviation (SD), median, minimum, and maximum for continuous variables and frequency counts, percentages, and exact 95% binomial confidence intervals (Clopper-Pearson) for proportions for categorical variables. Likewise, analyzed data sets of the registry have been described before [15].

In the neurological/spinal cohort, all patients who received Hemopatch^®^ were included in the safety analysis set and all patients with a postoperative hemostasis assessment were included in the full analysis set.

Post hoc evaluation of the neurological/spinal cohort

**Figure 1:**
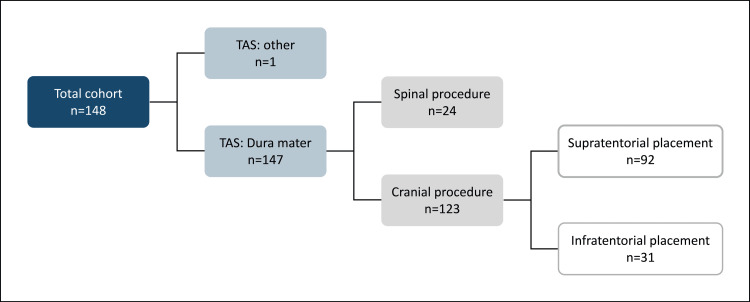
Sub-cohorts in neurological/spinal registry cohort. TAS = target application site

For a more detailed post hoc evaluation of the neurological/spinal cohort, this cohort was split into sub-cohorts. Based on the indication, procedure description, and the TAS, the documented cases were divided into a spinal and a cranial sub-cohort. For cranial procedures, using the same approach, the cases were further stratified into sub-cohorts with supratentorial or infratentorial Hemopatch^®^ application (Figure 1).

Documentation of the TAS provided information on (1) whether Hemopatch^®^ was placed on the dura and (2) whether Hemopatch^®^ was primarily used to achieve hemostasis or sealing, i.e. in combination with other documented parameters such as achievement of watertight intraoperative closure.

As per definition, watertight intraoperative closure of the dura was achieved if no CSF leakage was detected after inspection according to the local standard of care (e.g. Valsalva maneuver). Intraoperative watertight closure was documented in the eCRF as yes, no, or not applicable (N/A).

In the neurological/spinal cohort, the incidence of postoperative CSF leakages was documented during four weeks of postoperative follow-up (external or internal accumulation, including pseudo meningocele). The incidence of CSF leakages was therefore determined for the defined sub-cohorts.

## Results

Characteristics of the neurological/spinal cohort

Four centers from two countries contributed cases to the neurological/spinal cohort: two from the Czech Republic and two from Spain, see Appendices. The number of cases per center ranged from 1 to 59.

The neurological/spinal cohort comprised 148 patients, adults and children; the mean age was 44.1 years (SD = 22.4 years, Table 1). Patient characteristics of all patients included in the registry have been published in detail before [15].

**Table 1:** Patient characteristics of the neurological/spinal cohort (full analysis set). ^a^ Minimally invasive surgery included cranial biopsies, mini-craniotomies, or craniotomies with endoscopic intervention or biopsies ^b^ Only patients with comorbidities CCI = Charlson Comorbidity Index Adapted from [15]

Number of patients	Neurological/spinal
	(n = 148)
Age, years	
Mean (SD)	44.1 (22.4)
Median (range)	48.5 (1-84)
Gender	
Male, n (%)	63 (42.6)
Female, n (%)	85 (57.4)
Surgical approach	
Open, n (%)	139 (93.9)
Minimally invasive^a^, n (%)	9 (6.1)
Patients with comorbidities, n (%)	3 (2.0)
CCI^b^, mean (SD)	4.00 (1.73)

Intra- and postoperative effectiveness of Hemopatch^®^ in the neurological/spinal cohort

In the neurological/spinal cohort, hemostasis within two minutes was achieved in 109 patients (73.6%) and within three minutes in the remaining 39 patients (26.4%). Hemostasis was achieved before closure in all cases and no rebleeding was documented. CSF leakage was reported in 11 cases (7.4%). In most procedures (128; 86.5%) one patch was used to support a watertight closure of the dura [15]. In one case, Tisseel fibrin sealant (Baxter, Vienna, Austria) was used in addition to Hemopatch^®^.

Detailed results of intra- and postoperative effectiveness of Hemopatch^®^ for all cohorts in this registry have been reported earlier [15].

Post hoc evaluation of the neurological/spinal cohort

Of the 148 patients in the neurological/spinal cohort, 123 patients had cranial procedures and 24 underwent spinal procedures (Figure 1). In one spinal procedure, Hemopatch^®^ was used solely for hemostasis on an extended wound surface after the resection of a sarcoma. Hence, in this case, the TAS was not the dura. Due to the large size of the wound, six patches were used. No other patient received more patches.

Use of bicarbonate

The eCRF did not require bleeding and/or grade of bleeding at the TAS to be recorded. However, according to the instructions for use, bicarbonate solution could be used in the absence of blood or body fluid to moisten the TAS before Hemopatch^®^ placement. This ensures a moist and slightly alkaline environment and improves the polymerization of the PEG [16]. The use of bicarbonate was recorded in the eCRF. One center contributed 41 cases to the cohort (27.7% of the 148 cases); bicarbonate was applied in all 41 of these cases. Bicarbonate was not used in any of the remaining 107 cases.

Hemopatch^®^ in cranial procedures

In all 123 cranial patients, the documented TAS was the dura. In three patients of the supratentorial sub-cohort, the intraoperative water tightness was documented as “not applicable” (N/A), and Hemopatch^®^ was used for the main purpose of epidural hemostasis. A postoperative CSF leak did not occur in any of these three cases (Table 2).

**Table 2:** Intra- and postoperative outcomes of procedures (full analysis set). N/A = not applicable * Product was applied to an intact dura for hemostasis ** Watertight approximation of the dura with sutures was not achieved before the application of Hemopatch®

Procedure	Placement	Intraoperative result	n	Postoperative CSF-leakage (up to four weeks)	
				n	%
Cranial	All	All	123	9	7.3
Cranial	Supratentorial	All	92	5	5.4
		Watertight	89	5	
		N/A	3*		
Cranial	Infratentorial	All	31	4	12.9
		Watertight	30	3	
		Unknown	1	1	
Spinal	All	All	24	2	8.3
		Watertight	11	2	
		N/A**	13	0	

One patient in the infratentorial sub-cohort was operated on because of a schwannoma. In this case, watertight closure of the dura was not assumed as it was documented in the eCRF as “unknown”. This patient suffered from a CSF leak during the four weeks of postoperative follow-up (Table 2).

Watertight closure of the dura was achieved in 119 patients of the cranial sub-cohort (89 from the supratentorial sub-cohort and 30 from the infratentorial sub-cohort). During the four-week follow-up period, a postoperative CSF leak was documented in eight of these 119 patients. For one patient from the infratentorial sub-cohort, the occurrence of a postoperative CSF leak was stated as “unknown”, since the patient died due to malignant brain edema less than a week after the index surgery (Table 2). The incidence of postoperative CSF leaks after four weeks in patients where intraoperative watertight closure was achieved was 6.8% (eight out of 118 patients). In three of these cases, a pre-existing CSF fistula was repaired during the index surgery.

The main reason for Hemopatch^®^ application in cranial procedures was the prevention of CSF leakage. Overall, the postoperative CSF leakage rate within four weeks in patients where the product was used as a dural sealant was 7.6% (nine out of 119).

Hemopatch^®^ in spinal procedures

The spinal cohort comprised 24 patients with spinal dura closure, who were recruited from three different neurosurgical departments. In 13 out of 24 patients, intraoperative watertight closure of the dura could not be achieved with a suture. In the remaining 11 patients, primary watertight closure was documented. Of these 24 patients, two (8.3%) developed postoperative CSF leakage during the four-week follow-up. Remarkably, in both patients, an intraoperative watertight closure was achieved during the index surgery; both patients underwent revision surgery due to a pre-existing CSF fistula (Table 2).

Safety

As assessed in the safety analysis set, no allergic reactions occurred in any of the registry´s patients [15]. In the neurological/spinal cohort, two serious adverse events of particular interest were observed but not classified as related to the study product. One patient experienced severe local infection. This subdural empyema was located at the site of intervention following the craniotomy of a tumor situated in the left parietal lobe. The other patient experienced a deep wound infection, characterized by suppuration through the surgical wound (with fever and malaise) after repair surgery of a CSF fistula.

As mentioned above, one patient was lost to follow-up one week after the index surgery due to death based on the underlying pathology and unrelated to the product application.

## Discussion

The results from the neurological/spinal cohort of the Hemopatch^®^ registry show the effectiveness and safety of Hemopatch^®^ as a hemostatic sealant in neurosurgery. This is in line with previous observations [1,4,10,12-14].

Intra- and postoperative effectiveness and safety of Hemopatch^®^

A detailed discussion of the intra- and postoperative effectiveness of Hemopatch^®^ compared to other products as well as of the safety of Hemopatch^®^ in this registry has been published previously [15]. In the neurological/spinal cohort, hemostasis was achieved within two minutes in 73.6% of the patients and in all patients after three minutes. No rebleeding was documented. Only limited data are available from other publications reporting the time to hemostasis in neurological or spinal surgery when Hemopatch^®^ was applied. Studies reporting comparable outcomes for other types of surgery documented hemostasis within two to three minutes in more than 90% of the patients [17,18]. However, since these studies report on general, cardiac, pulmonary, and urological interventions, their results are of limited comparability to the results of our registry sub-cohort.

Overall, CSF leakage was reported in 7.4% of the patients in the neurological/spinal cohort [15]. This is in line with previously reported rates of CSF leakage, which ranged from 0 to 34.6% [1-3,5-10,12].

Of the two adverse events of special interest observed in our neurological/spinal cohort, neither was related to the use of Hemopatch^®^. Similarly, Montano et al. used Hemopatch^®^ in combination with fibrin sealant in cranial and spinal procedures and did not observe any adverse reactions [1]. The same holds true for Schebesch and Brawanski, who observed neither adverse events nor allergic reactions in response to Hemopatch^®^ in their series of cranial procedures [4]. Likewise, Nowak et al. concluded from two clinical observations that Hemopatch^®^ can be used safely for dural sealing [13,14].

Post hoc evaluation of CSF leakage rates after cranial procedures

In cranial procedures, we observed a postoperative CSF leakage rate of 7.3% (nine out of 123; Table 2). Three previously published smaller series describe lower CSF leakage rates following cranial procedures.

Schebesch and Brawanski [4] reported 22 consecutive patients undergoing cranial procedures, in which Hemopatch^®^ was used as the only sealant to cover the suture of the dura in order to achieve a watertight closure. None of these patients had undergone a cranial neurosurgical procedure before. The median follow-up was three months. In one patient, large gaps of >3 mm were present after suture approximation of the dura, resulting in a postoperative CSF leak (one out of 22; 4.5%) [4].

Nowak et al. published a series of 34 prospectively analyzed patients, in which Hemopatch^®^ was used to cover a suture line or a biological graft (muscle flap, periosteum, or heterologous pericardium) to achieve watertight dural closure. In their follow-up, they observed a CSF fistula rate of 5.9% (2/34 patients) [13].

Montano et al. described a small series of eight patients receiving a cranial procedure. During the follow-up, no CSF leakage was observed. However, additional fibrin sealant was used in all cases to seal the edges of Hemopatch^®^ and ensure optimal dural sealing [1].

The additional use of fibrin sealant by Montano and colleagues as well as the smaller number of cases in all three studies might contribute to the lower rates of postoperative CSF leaks observed. Nevertheless, the observations of Schebesch and Brawanski [4], Montano et al. [1], and Nowak et al. [13] support the effectiveness and safety of Hemopatch^®^ in dural closure.

Two other studies describe postoperative CSF leakage rates following cranial interventions that are comparable to the rate of 7.3% reported here (Table 2).

Sánchez Fernández and Rodríguez-Arias reported complex dural reconstructions including the posterior fossa. In their cohort study, they compared procedures in which the surgeon decided to use either Hemopatch^®^ as an adjunct during reconstruction or other products such as glues, dural substitutes, etc. The repair procedure employing Hemopatch^®^ led to a CSF leakage rate of 7.8% vs. 30.4% for the procedures that did not employ Hemopatch^®^ [12]. This supports the effectiveness of Hemopatch^®^ in preventing CSF leakage.

Nowak et al. also compared the effectiveness of Hemopatch^®^ as a dural sealant in posterior fossa neurosurgery to Tachosil^®^. They used Hemopatch^®^ in 40% of the cases for complex reconstructions and in 60% for sealing the dura suture. Overall, their CSF leakage rate in the cohort with Hemopatch^®^ was 10.3%; (four out of 39). In the Tachosil^®^ cohort, this product was only used in 9.4% for complex reconstructions and in more than 90% for sealing dura suture lines. The CSF leak rate was 8.0% in this cohort (18 out of 224) [14].

Post hoc evaluation of CSF leakage rates after spinal procedures

In the 24 spinal procedures of our registry, a postoperative CSF leak was observed in two cases (8.3%, Table 2).

So far, spinal procedures with Hemopatch^®^ have only been reported in the series of Montano et al., who included 14 patients undergoing spinal procedures in addition to the eight patients receiving a cranial procedure described above. In their spinal procedures, Hemopatch^®^ was applied to cover either incidental durotomies (nine cases, without suture repair), or additional suture holes after suture closure of the dura following resection of intradural tumors (five cases). In none of the 14 patients undergoing spinal surgery was a CSF leak observed during the postoperative follow-up. However, as described above for all the patients of Montano et al., fibrin sealant was used in addition to seal the edges of Hemopatch^®^ [1]. In the cohort analysis presented here, in only one case was Tisseel fibrin sealant used in addition to Hemopatch^®^. The consistent additional use of fibrin sealant and the low number of cases might explain the lower CSF leakage rate observed in the series by Montano et al.

The results of other studies reported a range from 3% to 20% after removal of an intradural spinal tumor [19-21] and 1-21% for incidental durotomy closure [22-24], and compare well with the rate of 8.3% CSF leakage in our registry.

Role of pre-existing CSF fistula

In our registry, two documented cases of postoperative spinal CSF fistula appeared in patients who underwent repair of a pre-existing CSF fistula. It is worth noting that in both patients, watertight closure of the dural repair with Hemopatch^®^ was initially achieved during the index surgery. The repair of existing CSF fistulas can be challenging due to various factors such as scarring, low vascularity of the surrounding tissues, shortage of dura to close the leak, and the difficulty of identifying the exact position of the leak in an inflammatory sub-acute wound [25]. Alexander et al. reported on a series of 34 cases of CSF fistula repair. Their success rate was 97% for a single endoscopic procedure. They concluded that the success of CSF fistula repair depends on an accurate pre-operative evaluation and surgical techniques [26].

Limitations

Limitations of the Hemopatch^®^ registry have already been stated in detail [15]. Briefly, the most important limitations were the lack of a control group as well as the lack of documentation of bleeding grade, e.g. according to the VIBe Scale (Validated Intraoperative Bleeding Scale) [27] before the application of Hemopatch^®^. In addition, documentation of the amount of CSF entering the drainage system at postoperative follow-up would be useful to further define effectiveness. Furthermore, due to its dual properties as a “sealing hemostat” and the fact that bleeding grades were not recorded before Hemopatch^®^ application, it was sometimes impossible to determine whether the main purpose of applying the product was hemostasis or sealing. Moreover, the conditions under which Hemopatch^®^ was applied may have varied widely because of the multicenter design and large sample size for the entire spine and the fact that supra- and infratentorial regions were included. However, the purpose of this registry was to investigate general efficacy and safety under real-world conditions.

## Conclusions

In this registry, we had the opportunity to analyze the outcomes of Hemopatch^®^ use in neurosurgery in a relatively large cohort including 148 patients. Watertight closure of the dura was virtually the exclusive reason for the application of Hemopatch^®^. This objective was accomplished in the majority of patients, and we observed no serious adverse events related to the use of Hemopatch^®^. The data obtained from the neurological/spinal cohort of our registry, therefore, confirm that the clinical use of Hemopatch^®^ as a dural sealant is safe and effective.

## Appendices

Surgeons contributing cases to the neurological/spinal cohort of the registry

Brno, Czech Republic: Radim Jančálek, Peter Solar

Madrid, Spain: Jose Asencio

Ostrava-Poruba, Czech Republic: Tomas Hrbac, Lukas Krska, Silvia Szathmáryova

Sevilla, Spain: Juan Martos, Marta Gonzalez Pombo, Javier Marquez-Rivas
